# A Hexa-Band Metamaterial Absorber for S, C, X, and Ku Band Applications

**DOI:** 10.3390/s26031040

**Published:** 2026-02-05

**Authors:** Muhammad Adnan Sultan, Inzamam Ahmad, Ashfaq Ahmad, Young-Jin Kim, Dong-You Choi

**Affiliations:** 1 Department of Electrical Engineering, Abasyn University Peshawar, Peshawar 25000, Pakistan; ibenesultan@gmail.com; 2 Department of Telecommunication Engineering, University of Engineering and Technology Mardan, Mardan 23200, Pakistan; inziahmad950@gmail.com; 3 Information and Communication Engineering, Chosun University, Gwangju 61452, Republic of Korea; ashfaquetb11@gmail.com; 4 Department of e-Sports Industry, Chosun College of Science & Technology, Gwangju 61543, Republic of Korea

**Keywords:** wide bandwidth, metamaterial, polarization-insensitive, impedance matching, microwave absorber

## Abstract

A compact and polarization-insensitive hexa-band metamaterial absorber (MMA) is designed, fabricated, and experimentally validated for S, C, X, and Ku band applications. The proposed unit cell consists of two square rings, two hexagonal rings, and two diamond-shaped copper resonators printed on an FR-4 dielectric substrate with a thickness of 1.6 mm. The structure exhibits six distinct absorption peaks at 2.178, 5.484, 8.391, 11.811, 15.858, and 18.689 GHz, with corresponding absorptivities of 99%, 98%, 99%, 99%, 99%, and 97%, respectively. The compact unit cell of 12.5 × 12.5 mm^2^ achieves high absorption efficiency due to strong electromagnetic coupling among the resonators. Simulated and measured results show strong agreement, confirming the accuracy of the design. Owing to its four-fold symmetric geometry, the absorber maintains stable performance under varying polarization angles and incidence angles up to 60° for both TE and TM polarizations. The electric field, magnetic field, and surface current distributions are analyzed to explain the absorption mechanism at each resonant frequency. The proposed MMA demonstrates multiband functionality, angular stability, and high absorptivity within a simple and low-cost design, making it a promising candidate for stealth, air traffic control, and satellite communication applications.

## 1. Introduction

Metamaterials are artificially engineered materials composed of periodically arranged metallic and dielectric components that exhibit exceptional electromagnetic properties not found in naturally occurring materials, such as negative refractive index, negative permittivity, negative permeability, and reversed phase velocity [1,2]. Owing to these unique properties, metamaterials have been widely utilized in microwave and electromagnetic applications, including tunable smart surfaces [3], filters [4], superlenses [5], antenna miniaturization [6], and absorbers [7].

The concept of the perfect metamaterial absorber (MMA) was first introduced by Landy et al. in 2008 [8], which triggered extensive research interest in this field. MMAs are designed to absorb most of the incident electromagnetic (EM) energy by minimizing both reflection and transmission. For an ideal MMA, perfect absorption is achieved when the MM absorbers are materials that absorb the majority of electromagnetic (EM) waves when they go through them. As a result, the MM absorber’s transmission coefficient is minimal, while the reflection coefficient is extremely low [9]. As a result, MM absorbers often have either a negative permittivity or a negative permeability, or both [10]. The metamaterial absorber solves the limitations that traditional microwave absorbers have in real-world applications.

Conventional microwave absorbers often face challenges such as large thickness, narrow bandwidth, structural fragility, and fabrication complexity [11]. In contrast, MMAs overcome these limitations by offering ultrathin, lightweight, and highly efficient designs [12]. MMA has been presented in a variety of forms, each with unique characteristics like polarization independence, and a wide oblique incidence angle. EM-based metamaterial absorbers are modeled according to the concept that the surface impedance meets free space impedance at optimal frequency [13]. When the structure’s Zin is equal to the free space’s impedance, the structure absorbs the incident wave fully, resulting in perfect absorption [14]. Patch and ground of absorbers are generally constructed of annealed copper, but the substrate material is the most significant aspect when building a metamaterial absorber [15]. The dielectric characteristics of various substrates are utilized to achieve the required absorption of the EM waves.

Among various substrate materials, FR-4 is frequently used in MMA fabrication due to its low cost, suitable dielectric properties, and easy availability [16]. The potential applications of MMAs extend across multiple domains, including photodetectors, sensors, solar photovoltaic systems, stealth technology, bolometers, infrared camouflage, and thermophotovoltaic devices [17,18].

Several polarization-independent multiband MMAs have been proposed in recent years. For instance, G. Wu et al. (2024) presented a ultrabroad band MMA comprising four metallic resonators on a 1.6 mm thick FR-4 substrate, exhibiting polarization insensitivity for both normal and oblique incidences [19]. Singh et al. demonstrated dual- and triple-band ultrathin conformal metamaterial absorbers exhibiting polarization insensitivity and wide angular stability, highlighting their suitability for practical electromagnetic compatibility and stealth-related applications [20]. Wang et al. (2019) developed a five-band MMA using L-shaped and slanted bar resonators, achieving absorption peaks at 3.90, 6.16, 8.02, 10.61, and 12.42 GHz with absorption rates exceeding 84% [21]. Dai et al. proposed a broadband MMA based on circular patches demonstrating polarization insensitivity and wide angular response [22]. Edries et al. (2020) reported a compact four-band MMA with absorptivity above 93% at 2.248, 2.878, 4.3, and 5.872 GHz, also exhibiting polarization-independent behavior [23]. Similarly, Hannan et al. proposed a fractal-based MMA for multiband operation, achieving absorption peaks at 11.23, 14.18, 17.37, and 19.18 GHz with polarization insensitivity for both normal and oblique incidences [24].

In this paper, a novel, compact, low-profile, and polarization-insensitive hexa-band metamaterial absorber is proposed for microwave applications. The unit cell consists of six metallic rings, two square, two hexagonal, and two diamond-shaped, that collectively produce six distinct absorption peaks at 2.178, 5.484, 8.391, 11.811, 15.858, and 18.689 GHz, with corresponding absorption rates of 99%, 98%, 99%, 99%, 99%, and 97%, respectively, covering the S, C, X, and Ku frequency bands. The unit cell has dimensions of 12.5 × 12.5 mm^2^ and employs a 1.6 mm thick FR-4 dielectric substrate between the resonator layer and the ground plane.

The polarization characteristics of the proposed MMA were analyzed for both normal and oblique incidences, and a parametric study was performed to optimize the resonant frequencies. The absorption mechanism at different frequency bands is explained through surface current, electric field, and magnetic field distributions. The proposed MMA demonstrates high absorptivity, polarization insensitivity, and compact geometry, making it a strong candidate for practical applications in stealth technology, communication systems, and sensing platforms.

## 2. Design

**Figure 1:**
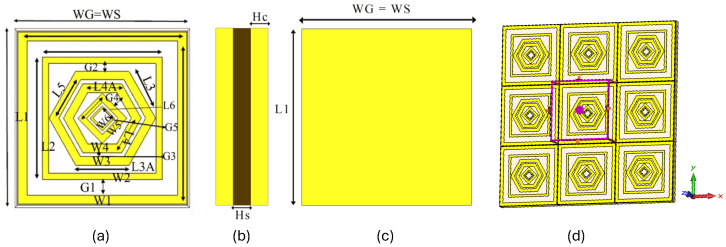
Geometrical configuration of the proposed metamaterial absorber (MMA). (**a**) Unit cell geometry, (**b**) Side view geometry, (**c**) Back view geometry, (**d**) 3 × 3 array of the proposed MMA showing boundary conditions.

The proposed MMA is illustrated in Figure 1, which shows the geometrical configuration of the metamaterial absorber unit cell. The side and back views of the unit cell are presented in Figure 1a and Figure 1b, respectively. Generally, a metamaterial absorber consists of three layers: a resonant metallic patch, a dielectric substrate, and a metallic ground plane. In the proposed design, the ground layer is completely covered with copper to prevent electromagnetic waves from transmitting through the structure. The top layer is also made of copper with a conductivity of $5.8\;\times \;10^{7}\;S/m$ and with the thickness of 0.035 mm to ensure high electrical conductivity. The intermediate dielectric layer is composed of FR-4 with a relative permittivity of $\varepsilon_{r}\;=\;4.4$ and a thickness of 1.6 mm. The dimensions of the resonators were determined through a trial-and-error optimization process to achieve the desired multi-band absorption response. The physical parameters of the proposed absorber are listed in Table 1. It should be noted that WG denotes the periodic unit-cell length, including edge clearance for boundary conditions, whereas L1 corresponds only to the outer square resonator length. Therefore, WG is intentionally chosen slightly larger than L1 to ensure proper periodicity and fabrication tolerance.

Accurate boundary conditions are essential for obtaining reliable simulation results. In this work, periodic unit-cell boundary conditions are applied along the *x*- and *y*-directions, while the *z*-direction is defined as open. These conditions are implemented using Floquet modes, which are equivalent to waveguide port excitations. Full-wave electromagnetic simulations are carried out using ANSYS HFSS 2021 R1 over a frequency range of 0–20 GHz. The solver employs the finite element method (FEM) to accurately evaluate the absorber performance, as illustrated in Figure 1d.

**Figure 2:**
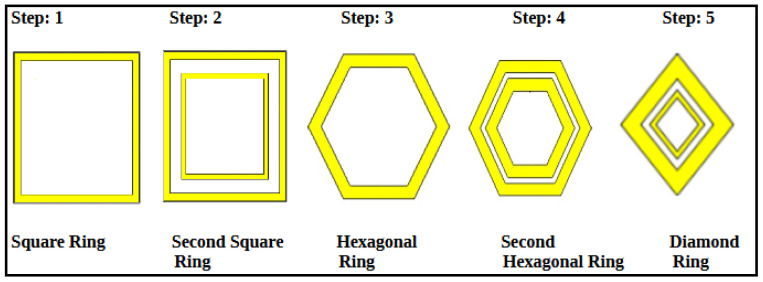
Step-by-step design of the unit cell (Steps 1–5).

**Figure 3:**
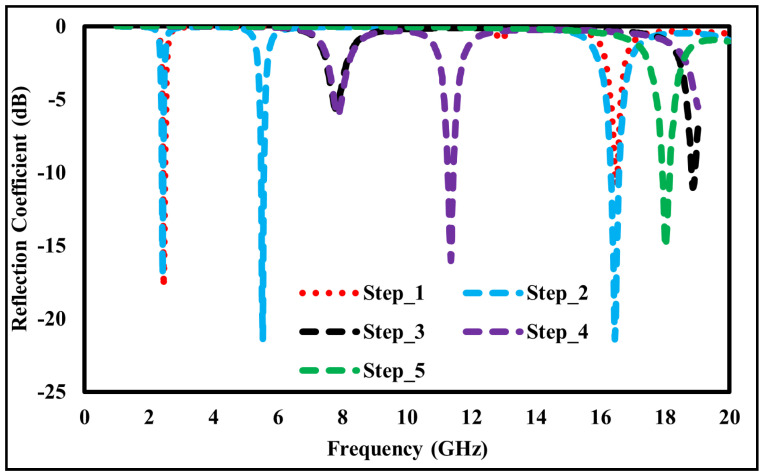
Step by Step Reflection coefficient of the unit cell absorber.

**Figure 4:**
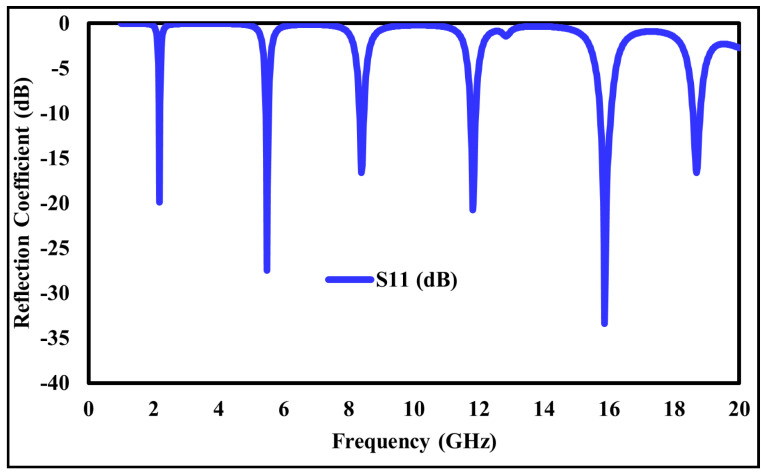
Reflection coefficient of the unit cell absorber.

The step-by-step design evolution of the proposed metamaterial absorber (MMA) unit cell is shown in Figure 2. The top metallic layer consists of six resonant elements: two square rings, two hexagonal rings, and two diamond-shaped rings. Each resonator is responsible for a distinct absorption band. Specifically, the outer square ring produces a resonance at 2.178 GHz, while the inner square ring generates resonances at 5.484 and 11.811. The two hexagonal rings correspond to resonances at 8.391 GHz and 15.858 GHz, whereas the diamond-shaped rings generate a high-frequency resonance at 18.689 GHz, as shown in Figure 3. Although individual resonators do not achieve perfect absorption, their combined configuration enables strong mutual coupling, significantly enhancing the overall absorption performance, as depicted in Figure 4.

The operating mechanism of the proposed metamaterial absorber (MMA) is governed by multi-resonant LC behavior and strong electromagnetic coupling among the nested resonators. Each metallic ring functions as an individual LC resonator, where the inductive component originates from circulating surface currents, while the capacitive component primarily arises from the inter-element gaps and the dielectric substrate. The geometrical dimensions of the resonators are carefully optimized so that their corresponding resonance frequencies are distributed across the S-, C-, X-, and Ku-bands.

**Figure 5:**
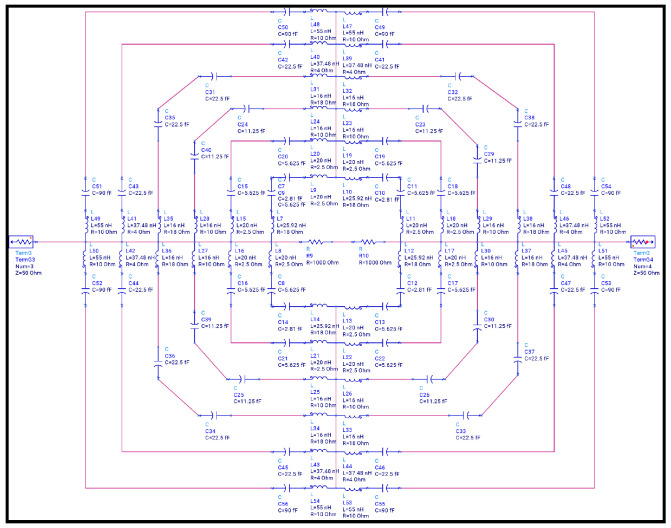
Equivalent circuit model of the unit cell. Blue represents the RLC components, and the red lines represent the conducting wires.

**Figure 6:**
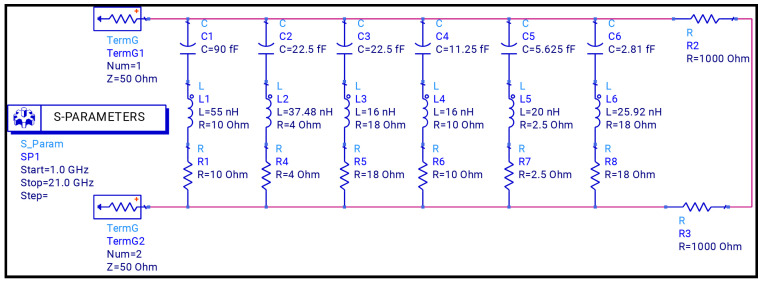
Transmission Line Model for Unit Cell.

**Figure 7:**
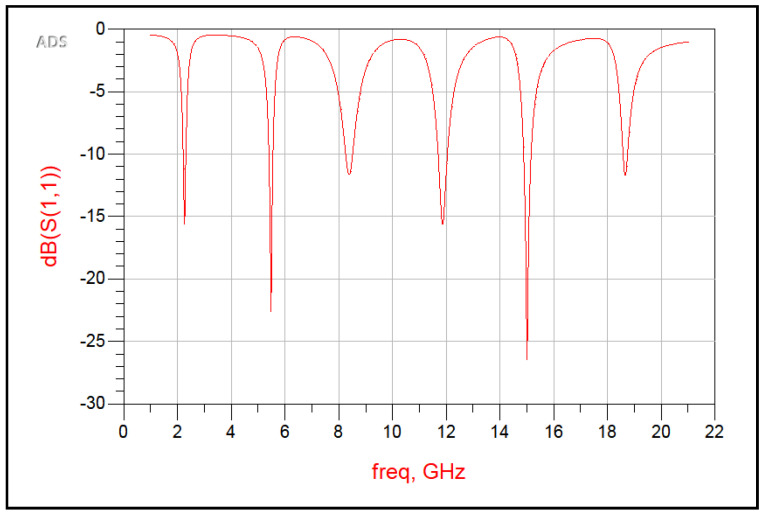
Frequency of Equivalent Circuit Model.

When the resonators are placed in close proximity, strong near-field interactions occur, leading to the formation of hybridized resonant modes. These coupled modes enhance impedance matching between the absorber and free space ($Z\;\approx \;Z_{0}\;=\;377\;\Omega$), resulting in multiple high-absorption peaks over the targeted frequency spectrum. The strong electromagnetic coupling is mainly attributed to the compact spatial arrangement of the resonant rings on the top metallic layer. Upon interaction with an incident electromagnetic wave, each resonator supports a localized LC resonance; due to the small inter-resonator spacing, the associated electric and magnetic fields overlap, forming coupled current loops. This synchronized interaction facilitates efficient energy redistribution among the resonators and leads to effective impedance matching at multiple discrete frequencies, thereby minimizing reflection and maximizing absorption. To further validate the electromagnetic behavior of the proposed unit cell, an LC equivalent circuit model is developed using Advanced Design System (ADS), as shown in Figure 5. In this model, each metallic resonator is represented by a series RLC branch, where the inductance (*L*) corresponds to the current flow paths, the capacitance (*C*) represents the capacitive coupling between adjacent metallic resonators and between the top metallic layer and the ground plane through the dielectric substrate, and the resistance (*R*) accounts for dielectric and ohmic losses. Mutual inductances (*M*) and coupling capacitances ($C_{c}$) are incorporated to model the near-field electromagnetic coupling between adjacent resonators. The complete equivalent circuit models of the proposed absorber are presented in Figure 5 and Figure 6, while the corresponding resonant frequencies obtained from these circuit models are illustrated in Figure 7. All equivalent circuits are designed and simulated using ADS.

The resonance behavior follows the classical LC relation:

(1) $$f_{r}\;=\;\frac{1}{2\pi \sqrt{LC}}$$

where *L* and *C* denote the effective inductance and capacitance, respectively. Maximum absorption occurs when the input impedance of the absorber matches the free-space impedance:

(2) $$Z_{\mathrm{in}}\;\approx \;Z_{0}\;=\;377\;\Omega$$

which minimizes reflection and ensures efficient electromagnetic energy dissipation within the lossy substrate and metallic resonators.

Table 2 presents the operating frequencies, 90% absorption bandwidths (A ≥ 0.9), absolute bandwidths, and fractional bandwidths of the proposed microwave absorber. Each absorption peak corresponds to a distinct resonant frequency, with the effective operating bandwidth defined as the frequency range over which the absorptivity remains equal to or greater than 90%. The operating bandwidth is determined by the difference between the upper and lower cutoff frequencies of the absorption band. This bandwidth is expressed directly in terms of frequency and is reported in MHz or kHz, providing a clear and practical measure of the absorber’s operational range. As shown, the absorber exhibits multiple resonances spanning from 2.178 to 18.689 GHz, covering the S, C, X, and Ku bands. The maximum absolute bandwidth of 0.40 GHz is achieved at 15.858 GHz, whereas the highest fractional bandwidth of 6.4% occurs at 2.178 GHz, demonstrating the multiband operation and high absorption performance of the proposed design.

In addition to multiband absorption, the proposed metasurface offers several advantages in terms of compactness and robustness. Unlike conventional designs where resonators operate independently, the integration of square, hexagonal, and diamond-shaped resonators enables intrinsic impedance matching at six distinct frequencies, achieving absorption levels of 97–99% within a compact $12.5\;\times \;12.5\;{\mathrm{mm}}^{2}$ unit cell. Compared to larger unit cells reported in the literature, this demonstrates effective miniaturization. Furthermore, the fourfold symmetric geometry ensures stable absorption performance for varying polarization states and oblique incidence angles up to 60°, making the design suitable for practical applications.

## 3. Absorber Theory and Metamaterial Behavior

Absorptivity quantifies the fraction of incident electromagnetic energy dissipated within a structure through dielectric and ohmic losses and should be clearly distinguished from mere reflection suppression. For a general electromagnetic structure, the absorptivity is defined as

(3) A(ω) = 1 − R(ω) − T(ω)

where $R\left(\omega \right)\;=\;|S_{11}{|}^{2}$ and $T\left(\omega \right)\;=\;|S_{21}{|}^{2}$ represent the reflected and transmitted power, respectively. This formulation is widely used to evaluate the absorption performance of metamaterial and frequency-selective surface absorbers.

In the proposed absorber, a continuous metallic ground plane is employed, which effectively suppresses transmission over the entire operating frequency range, such as, $|S_{21}{|}^{2}\approx 0$. Consequently, Equation (3) simplifies to

(4) $$A\left(\omega \right)\;=\;1\;-\;|S_{11}{|}^{2}$$

This indicates that the absorption performance is primarily determined by the reflection coefficient. It is important to note that a minimum in the reflection coefficient alone does not necessarily imply true electromagnetic absorption. Reflection minima may also arise from phase cancellation or impedance mismatch effects without significant energy dissipation. Genuine absorption occurs only when the incident electromagnetic energy is effectively coupled into the structure and dissipated through dielectric and ohmic losses. In the proposed design, the reflection minima coincide with strong electric and magnetic field confinement and pronounced surface current distributions within the resonant elements, confirming that the incident energy is dissipated inside the absorber rather than merely redirected.

The reflection coefficient can be related to the surface impedance of the absorber through standard microwave theory as

(5) $$\Gamma \left(\omega \right)\;=\;\frac{Z\left(\omega \right)\;-\;Z_{0}}{Z\left(\omega \right)\;+\;Z_{0}}$$

where Z(ω) denotes the surface impedance of the absorber and $Z_{0}\;=\;377\;\Omega$ is the free-space impedance. Perfect absorption is achieved when Z(ω) is matched to $Z_{0}$, resulting in minimal reflection.

Under the effective medium approximation, the surface impedance may be expressed in terms of effective material parameters as

(6) $$Z\left(\omega \right)\;=\;\sqrt{\frac{\mu_{r}\mu_{0}}{\varepsilon_{r}\varepsilon_{0}}}$$

where $\varepsilon_{0}$ and $\mu_{0}$ are the permittivity and permeability of free space, respectively, and $\varepsilon_{r}$ and $\mu_{r}$ denote the effective relative permittivity and permeability. For ground-backed absorber structures, these retrieved parameters do not represent intrinsic bulk material properties but instead provide qualitative insight into the excitation of electric and magnetic resonances that contribute to impedance matching.

For a physically rigorous description of the absorption mechanism, the normalized input impedance can be directly extracted from the scattering parameters as

(7) $$Z_{\mathrm{in}}\;=\;\sqrt{\frac{{\left(1\;+\;S_{11}\right)}^{2}\;-\;S_{21}^{2}}{{\left(1\;-\;S_{11}\right)}^{2}\;-\;S_{21}^{2}}}$$

which, for the proposed ground-backed absorber, reduces to a function of $S_{11}$ only. Near-perfect absorption is achieved when the real part of $Z_{\mathrm{in}}$ approaches unity and the imaginary part approaches zero, indicating effective impedance matching with free space and minimal reflection.

## 4. Simulation Results: Discussion and Analysis

**Figure 8:**
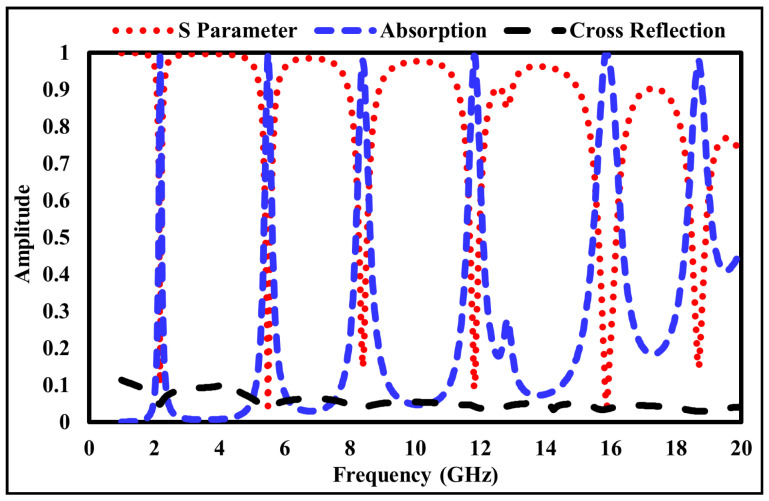
Simulated absorption and reflection characteristics of the proposed metamaterial absorber.

The reflection and absorption characteristics of the proposed metamaterial (MM) absorber are illustrated in Figure 8. The combined plot demonstrates that the designed structure exhibits six distinct absorption peaks and corresponding reflection minima at frequencies of 2.178, 5.484, 8.391, 11.811, 15.858, and 18.689 GHz. The respective absorption magnitudes are 99%, 98%, 99%, 99%, 99%, and 97%, while the corresponding reflection coefficients are −19.88, −27.45, −16.59, −20.73, −33.40, and −16.63 dB.

It is evident that as the reflection coefficient decreases (i.e., lower reflected power), the absorptivity increases, confirming efficient impedance matching between the absorber surface and free space. The absorption peaks fall within the S, C, X, and Ku microwave frequency bands, demonstrating the wideband and multiband absorption capabilities of the proposed design. Therefore, the absorber is a promising candidate for various microwave applications, including stealth, electromagnetic shielding, and radar cross-section reduction.

## 5. Effect of Structural Parameters

**Figure 9:**
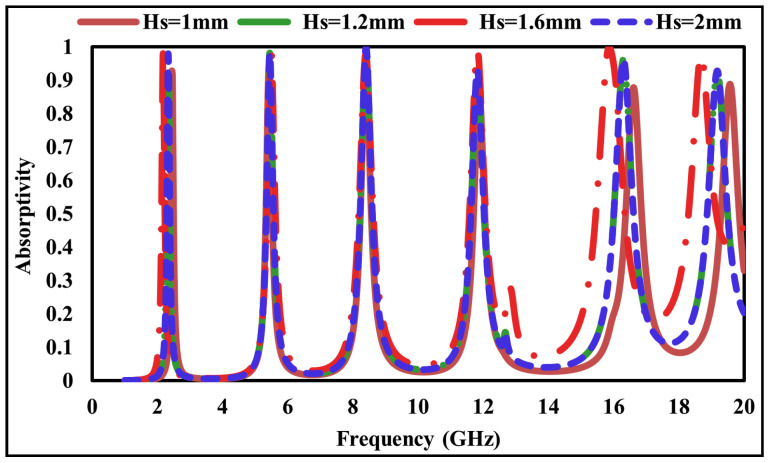
Absorption response of the proposed MMA at different substrate thicknesses.

FR-4 is used as the dielectric substrate in the proposed structure. To investigate the effect of substrate thickness on the absorption performance, several FR-4 thicknesses 1.0 mm, 1.2 mm, 1.6 mm, and 2.0 mm were analyzed. As shown in Figure 9, the absorption values of the unit cell exceed 84% for a 1.0 mm thick substrate, 88% for 1.2 mm, and 97% for 1.6 mm. For a 2.0 mm substrate, the absorption remains above 93%. These results indicate that the absorption characteristics vary with substrate thickness, and the optimal performance is achieved at a thickness of 1.6 mm.

**Figure 10:**
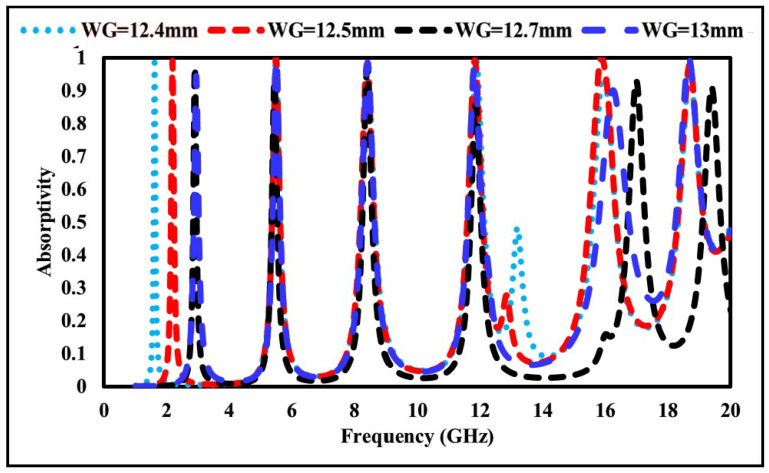
Absorption characteristics at different ground and substrate lengths.

To further analyze the influence of structural dimensions, a parametric study was performed by varying the ground and substrate lengths ($W_{G}$ and $W_{S}$). The corresponding absorption variations are presented in Figure 10. When $W_{G}$ and $W_{S}$ are set to 12.4 mm, absorption values of 98% at 1.69 GHz, 98% at 5.40 GHz, 96% at 8.44 GHz, 97% at 11.81 GHz, 92% at 16.02 GHz, and 97% at 18.72 GHz are achieved. Increasing $W_{G}$ and $W_{S}$ results in variations in absorptivity, particularly a reduction at higher frequencies. Parametric values of 12.5 mm, 12.7 mm, and 13.0 mm were analyzed, with maximum absorption obtained at $W_{G}\;=\;12.5\;\mathrm{mm}$.

**Figure 11:**
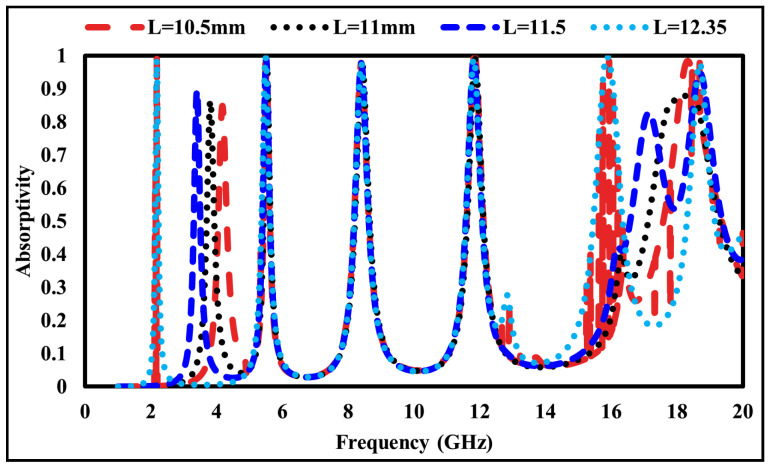
Absorption response at different lengths (*L*) of the first square ring.

A similar parametric analysis was conducted by varying the length of the first square ring ($L_{1}$) while keeping all other parameters constant, as shown in Figure 11. At $L_{1}\;=\;10.5\;\mathrm{mm}$, an absorption of 83% occurs at 4.192 GHz. Increasing $L_{1}$ to 11.0 mm and 11.5 mm shifts the resonance to 3.793 GHz and 3.394 GHz, with absorptions of 85% and 89%, respectively. The maximum absorption of 98% is achieved at 2.178 GHz when $L_{1}\;=\;12.35\;\mathrm{mm}$.

The main loss mechanisms in the proposed metamaterial absorber are ohmic losses in the metallic resonators and dielectric losses in the substrate. When the incident electromagnetic wave excites the localized LC resonances of the metallic rings, strong surface currents are induced along the conductive paths, forming closed-loop currents that dissipate energy as heat due to the finite conductivity of the metals (ohmic losses). At the same time, the alternating electric fields across the capacitive gaps polarize the dielectric substrate, causing dielectric loss that further converts the incident electromagnetic energy into heat. The combination of these two loss mechanisms ensures that the incident energy is effectively absorbed, resulting in the high absorptivity observed at the resonant peaks.

## 6. Absorbance Profile Under Polarization Angle Variations

**Figure 12:**
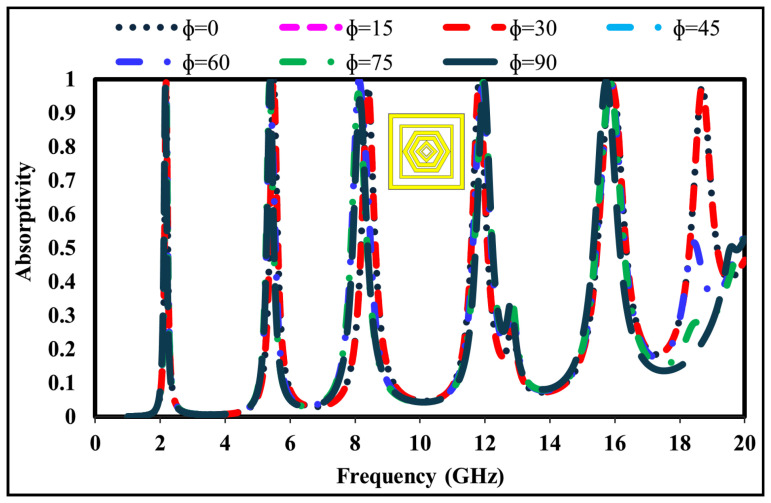
Simulated absorptivity of the proposed metamaterial absorber for different polarization angles (Φ).

Polarization insensitivity and angular stability are essential characteristics of high-performance electromagnetic absorbers. An ideal absorber should retain its absorption capability regardless of the polarization state of the incident electromagnetic wave. Figure 12 illustrates the simulated absorption response of the proposed structure for different polarization angles ($\Phi \;=\;0^{\circ },\;15^{\circ },\;30^{\circ },\;45^{\circ },\;60^{\circ },\;75^{\circ },\;90^{\circ }$) under normal incidence.

The results indicate that the peak absorptivity remains almost constant for all polarization angles, verifying the polarization-insensitive behavior of the design. This consistent response arises from the four-fold geometric symmetry of the metamaterial unit cell, which ensures identical absorption characteristics when the structure is rotated about the *z*-axis or within the xy-plane. Consequently, the proposed absorber demonstrates stable absorption performance under varying polarization conditions, a key requirement for practical microwave and radar applications.

## 7. Absorbance Profile Under Oblique Incidence Angle Variations

**Figure 13:**
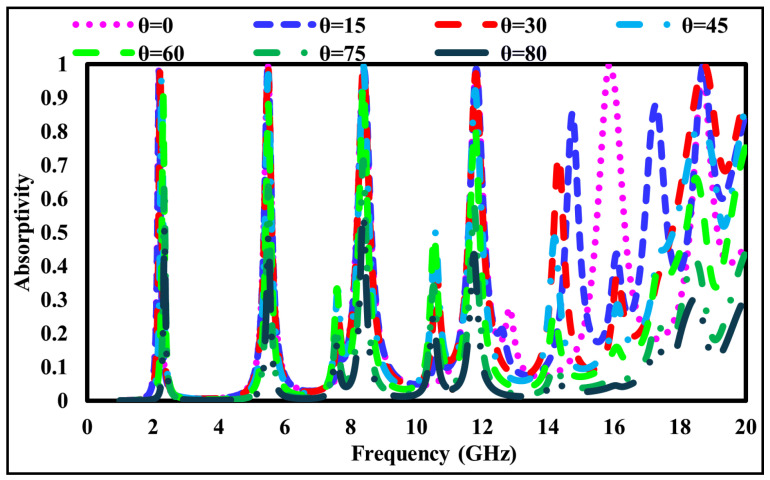
Absorptivity variation at different incident angles (θ) for TE-polarized waves.

**Figure 14:**
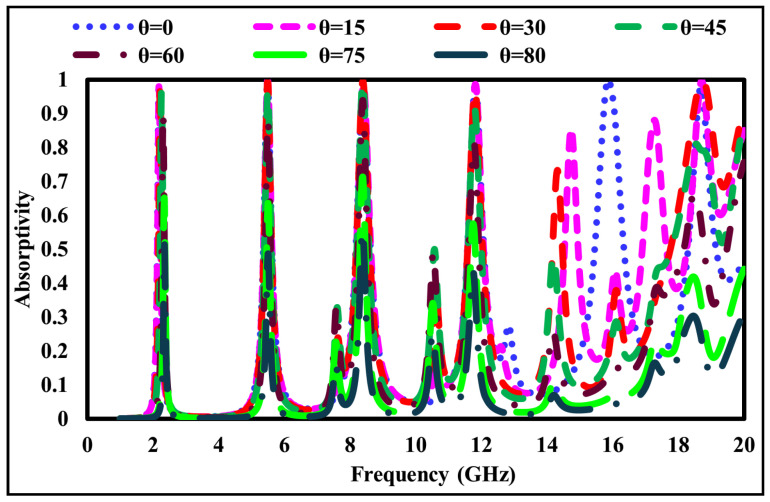
Absorptivity variation at different incident angles (θ) for TM-polarized waves.

In practical scenarios, electromagnetic waves may impinge on a surface at arbitrary angles. Therefore, the angular stability of the proposed six-band metamaterial absorber was evaluated under oblique incidence for both TE- and TM-polarized waves. The simulated absorptivity at incidence angles (θ) of $0^{\circ }$, $15^{\circ }$, $30^{\circ }$, $45^{\circ }$, $60^{\circ }$, $75^{\circ }$, and $80^{\circ }$ is presented in Figure 13 for TE polarization and Figure 14 for TM polarization.

As observed from these figures, the absorber maintains an absorptivity exceeding 85% up to an incidence angle of 60° for both polarization states, indicating robust angular stability within this range. Beyond 60°, a gradual degradation in absorption performance is observed, particularly at higher frequencies, where oscillatory behavior appears. These effects are attributed to increased inter-resonator coupling and the excitation of higher-order Floquet modes at extreme incidence angles. Nevertheless, the absorber remains effective over a wide angular range, which is consistent with the behavior of multiband frequency-selective surface absorbers. The practical relevance of each resonant mode is further assessed by evaluating the operating bandwidth at each absorption peak using the standard A ≥ 0.9 (90%) criterion. This behavior is governed by the coupled-resonator topology (square–hexagonal–diamond rings) and the intrinsic loss characteristics of the FR-4 substrate, which together determine the selectivity of each absorption band. Although absorption performance degrades at extreme incidence angles, several strategies may be employed to improve wide-angle stability in future designs, including the use of gradient-index or multilayer superstrates, lossy or magnetic loading to suppress higher-order modes, and geometric optimization of the resonant elements to reduce angle-sensitive current paths. These approaches provide a clear pathway for enhancing angular robustness in future work.

Overall, the presented analysis emphasizes both bandwidth performance and angular stability, demonstrating that the proposed absorber offers practical multiband absorption characteristics beyond isolated resonance peaks.

## 8. Absorption Mechanism

**Figure 15:**
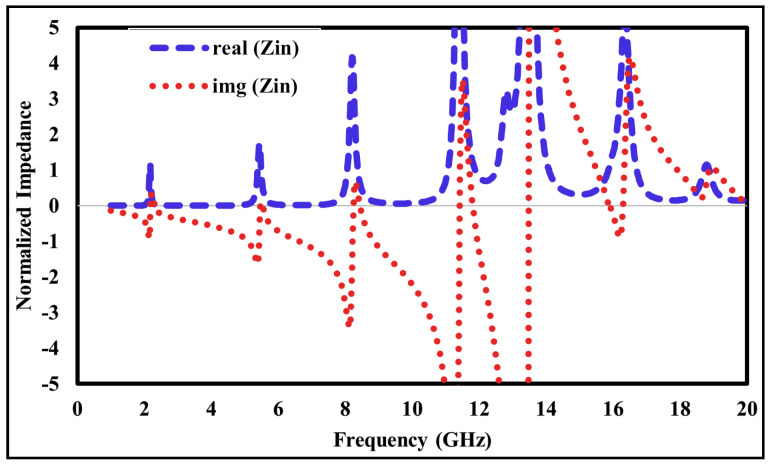
Simulated input impedance of the proposed absorber.

To investigate the absorption process of the unit cell absorber, the normalized input impedance, electric field, magnetic field, and surface current distribution have been analyzed at all six absorption peak frequencies. The real and imaginary components of the input impedance, normalized to the free-space impedance (377 + j0Ω), are shown in Figure 15. According to absorption theory, perfect absorption is entirely dependent on the input impedance of the structure. To achieve a high absorption ratio, the input impedance must be well matched to the free-space impedance. For verification, the input impedance characteristics were plotted over the entire frequency band. At resonance, the real component of the impedance is approximately unity, while the imaginary component is nearly zero.

**Figure 16:**
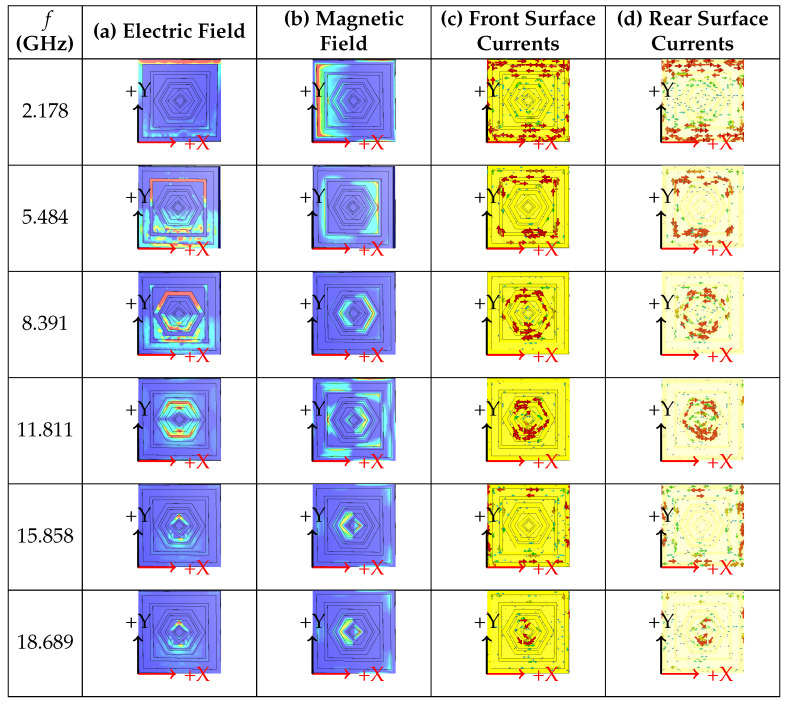
Instantaneous distribution of (**a**) electric field, (**b**) magnetic field, (**c**) front surface current, and (**d**) back surface current at 2.178, 5.484, 8.391, 11.811, 15.858, and 18.689 GHz.

The simulated electric field distributions at the resonant frequencies are shown in Figure 16a. In the diagrams, red indicates high electric field intensity, while blue represents low intensity. At 2.178 GHz, the electric field is concentrated at the top of the first square ring of the resonator. At 5.484 GHz, the field is localized in the upper part of the second square ring. For 8.391 GHz, the field intensity is strong at the top and bottom sections of the third hexagonal ring. At 11.811 GHz, the electric field is maximal at the upper and lower corners of the fourth hexagonal ring. At 15.858 GHz, the field is distributed across the first, second, and fifth/sixth rings of the structure. Finally, at 18.689 GHz, a strong electric field is coupled between the fifth and sixth diamond-shaped rings.

The magnetic field distributions are shown in Figure 16b. The magnetic field component is parallel to the *x*-axis, with high intensity along the +x and −x directions and weaker intensity along the +y and −y directions. At the resonance of 2.178 GHz, the magnetic field is strongly concentrated near the sidewalls of the first square ring of the absorber. Comparing the magnetic and electric fields, it is observed that the magnetic field is stronger in regions where the electric field is weaker, and vice versa, indicating a complementary distribution between the two fields. The surface current distributions on the front and rear surfaces are shown in Figure 16c and Figure 16d, respectively. On both the upper and lower surfaces of the absorber, distinct current patterns are observed. At 2.178 GHz, the current is maximized at the first square ring. At 5.484 GHz, the maximum current is observed at the second square ring. At 8.391 GHz, the current is concentrated in the first hexagonal ring, while at the 11.811 GHz absorption peak, it is concentrated in the second hexagonal ring. At 15.858 GHz, the surface currents are distributed across the entire structure, and at 18.689 GHz, the maximum current is localized in the diamond-shaped ring. It is observed that the surface currents on the upper layer are localized at different parts of the unit cell at different frequencies. Furthermore, the surface currents on the upper and lower metal layers are parallel but flow in opposite directions, forming closed current loops within the metamaterial absorber. These loops induce a magnetic field, which leads to energy dissipation in the structure and results in the observed high absorption. To clarify the physical mechanism underlying each absorption band, the dominant resonant mode corresponding to every absorption peak has been identified and analyzed. The electric field, magnetic field, and surface current distributions at each resonant frequency were re-examined, allowing a clear association between each absorption peak and its governing electromagnetic mode. The operating mechanism of the proposed absorber is summarized in Table 3.

## 9. Experimental Results

**Figure 17:**
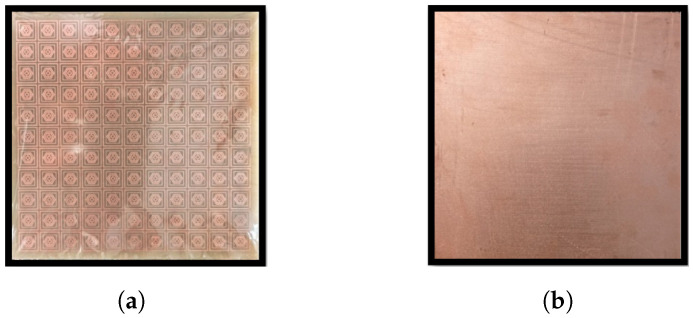
Experimental Verification of the Designed Structure (Fabrication): (**a**) Front View and (**b**) Back View.

**Figure 18:**
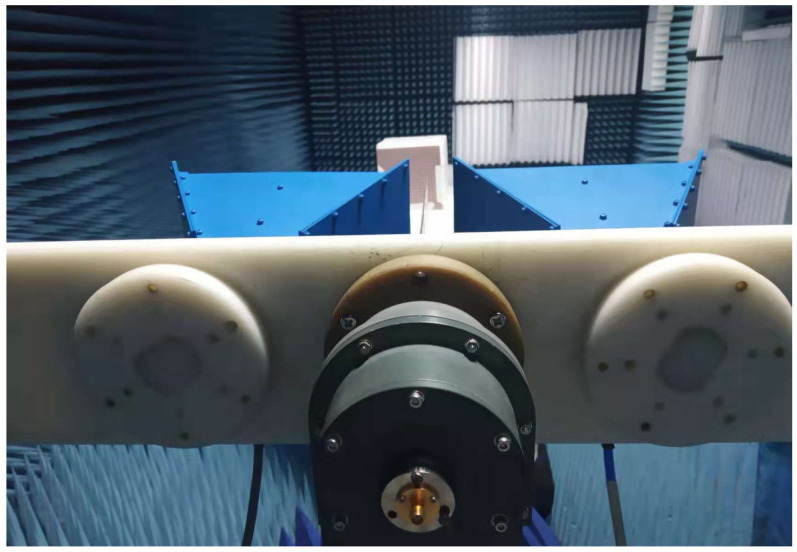
Practical setup for measuring the absorptivity of the metamaterial absorber. A VNA is connected to transmit (Tx) and receive (Rx) antennas, and the sample is placed on a holder. Absorptivity is calculated from $A\left(\omega \right)=1\;-\;|S_{11}{|}^{2}$.

The designed metamaterial absorber (MMA) was fabricated, and Figure 17 shows a photograph of the constructed prototype. The prototype consists of an 11 × 11 array of unit cells, resulting in an overall size of 152 × 152 mm^2^. A schematic of the experimental measurement setup is illustrated in Figure 18. The reflection characteristics of the fabricated prototype were measured using a standard free-space reflection measurement configuration, employing broadband horn antennas connected to an Agilent N5221A vector network analyzer (VNA). The antennas were positioned in the far-field region of the sample to ensure plane-wave illumination, with the separation distance chosen to satisfy the far-field condition for the lowest operating frequency.

**Figure 19:**
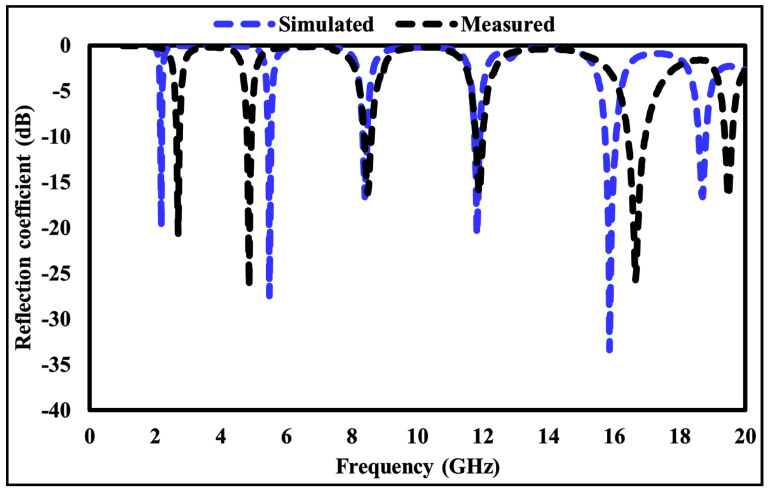
Simulated and measured reflection coefficient of the suggested metamaterial absorber.

**Figure 20:**
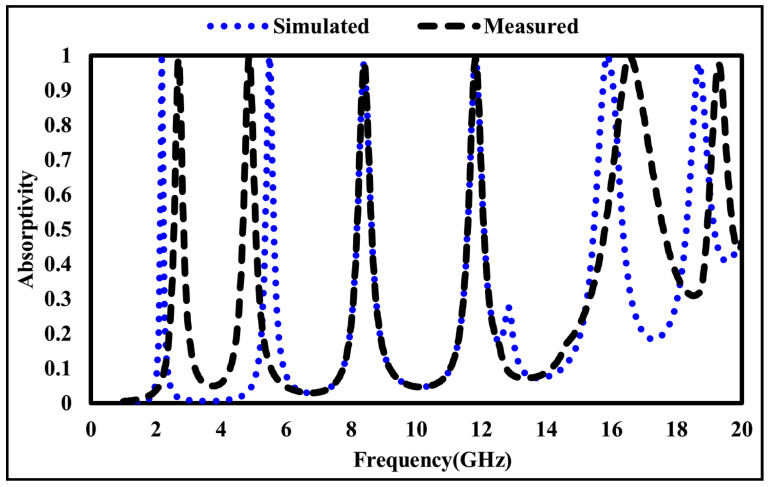
Simulated and measured absorptivity of the suggested metamaterial absorber.

Prior to measurement, a flat copper sheet with the same dimensions as the fabricated absorber was placed at the sample position and used as a reference reflector to calibrate the measurement system and establish the reference plane. The fabricated MMA was then positioned at the same location, and its reflection coefficient was measured under identical conditions. The reflection response of the proposed absorber was obtained by normalizing the measured reflected signal with respect to the copper reference. Since the absorber incorporates a continuous metallic ground plane, transmission is effectively suppressed, and the absorptivity was calculated using $A\left(\omega \right)\;=\;1\;-\;|S_{11}{|}^{2}$. The measured results were compared with the simulated responses, as shown in Figure 19 and Figure 20. Apart from a minor frequency shift, the measured and simulated results exhibit good agreement. The observed discrepancies are mainly attributed to fabrication tolerances, the finite size of the fabricated prototype, and measurement uncertainties.

Table 4 presents a performance comparison between the proposed absorber and recently reported metamaterial absorbers in the literature, considering parameters such as unit-cell dimensions, number of absorption bands, substrate thickness, and absorptivity. As evident from the comparison, the proposed design demonstrates notable advantages in terms of compact unit-cell size, high absorption efficiency, and an increased number of resonant frequency bands.

### 9.1. Case Study: EMI Suppression in Equipment Enclosures

To illustrate the potential practical applicability of the proposed hexa-band metamaterial absorber, a representative case study is considered in which the absorber is integrated inside a metallic equipment enclosure for electromagnetic interference (EMI) mitigation. Industrial and communication-grade electronic systems commonly exhibit undesired electromagnetic emissions in the 2–6 GHz frequency range, while high-speed digital electronics and radar-adjacent subsystems may generate leakage within the 8–12 GHz (X-band) region. If left untreated, such emissions can lead to internal coupling, signal degradation, or unintended radiation, potentially resulting in electromagnetic compatibility (EMC) compliance issues.

In this illustrative scenario, the proposed metasurface absorber is assumed to be applied as an internal lining on selected enclosure walls. The fabricated prototype, with a size of 152 × 152 mm^2^ (11 × 11 unit cells), is suitable for modular tiling, enabling scalable surface coverage within enclosures of varying dimensions. The absorption peaks at 2.178 GHz and 5.484 GHz coincide with frequency bands commonly associated with industrial controllers, RFID systems, and Wi-Fi interference, while the resonances at 8.391 GHz and 11.811 GHz correspond to emissions generated by high-speed clock harmonics and radar front-end leakage. Owing to the angular stability of the absorber up to 60° incidence, internally reflected waves originating from components and cabling are expected to experience effective absorption rather than re-radiation.

Compared to conventional EMI mitigation materials such as ferrite tiles or carbon-loaded foam absorbers, the proposed metasurface offers a significantly thinner profile (1.6 mm) and frequency-selective multiband absorption. This selective response enables targeted suppression of undesired resonances while minimizing broadband signal attenuation, thereby allowing coexistence with desired operational channels. Table 5 summarizes the correspondence between the absorber resonance frequencies and typical EMI sources encountered in metallic enclosures.It should be noted that this case study is illustrative in nature and is intended to demonstrate potential application scenarios rather than provide a quantitative enclosure-level EMI reduction measurement.

This case study highlights the suitability of the proposed absorber for frequency-selective EMI mitigation in compact enclosures. A detailed quantitative assessment, including system-level full-wave simulations and controlled chamber measurements, is identified as an important direction for future work to validate the enclosure-level EMI reduction performance.

### 9.2. Thermal, Environmental, and System-Level Considerations

Although the primary focus of this work is electromagnetic design and characterization, thermal stability, environmental robustness, and system-level integration are important considerations for practical deployment. Based on the material properties used in this study, the proposed absorber is expected to exhibit stable performance under typical operational conditions. FR-4 substrates are widely employed in microwave systems due to their relatively stable dielectric properties over a temperature range of approximately −10 °C to +85 °C and low moisture absorption (∼0.1–0.2%), while copper metallization exhibits negligible conductivity variation within this range.

For applications requiring extended environmental durability, protective strategies such as conformal coating, hydrophobic surface treatment, or encapsulation may be employed. Additionally, the absorber geometry can be readily adapted to alternative low-loss substrates (e.g., Rogers or PTFE-based laminates) to support outdoor or high-reliability applications. From a system-integration perspective, the proposed metasurface may be incorporated as a frequency-selective coating or back-surface lossy layer in antenna platforms, radar modules, or communication systems to suppress mutual coupling and reduce radar cross-section (RCS). Future experimental studies will focus on integrating the absorber with antenna and radar subsystems to evaluate its impact on radiation characteristics, sidelobe suppression, and electromagnetic coupling in realistic operational environments.

## 10. Conclusions

In this work, a compact and polarization-independent six-band metamaterial absorber has been designed, fabricated, and experimentally characterized for S-, C-, X-, and Ku-band applications. The proposed absorber consists of two square rings, two hexagonal rings, and two diamond-shaped rings, producing six distinct absorption peaks at 2.178, 5.484, 8.391, 11.811, 15.858, and 18.689 GHz, with corresponding absorptivities of 99%, 98%, 99%, 99%, 99%, and 97%, respectively. A 1.6 mm thick FR-4 substrate was employed for both simulation and fabrication, and the unit cell occupies a compact footprint of 12.5 × 12.5 mm^2^. Owing to its four-fold symmetric geometry, the absorber exhibits polarization-independent behavior and maintains high absorption performance for oblique incidence angles up to 60°. The absorption mechanism was investigated through normalized input impedance analysis, electric field distributions, and surface current responses, confirming that the observed absorption peaks arise from effective surface impedance matching and electromagnetic energy dissipation within the resonant elements. Experimental measurements performed on a fabricated prototype show good agreement with the simulated results, validating the proposed design. Due to its compact size, multi-band operation, angular stability, and simple planar configuration, the proposed absorber represents a promising candidate for frequency-selective electromagnetic applications such as stealth technology, electromagnetic interference suppression, air traffic control, and satellite communication systems.

## Figures and Tables

**Table 1:** Dimensions and parameters of the MMA.

Parameters	Size (mm)	Parameters	Size (mm)
WG	12.5	W1	0.65
L1	12.35	W2	0.4
L2	8.6	W3	0.72
L3	4	W4	0.72
L4	3	W5	0.45
L5	2.5	W6	0.15
L6	1.3	G1	1.88
G2	0.50	G3	2.55
G4	0.25	G5	0.60
L3A	3.5	L4A	2.5
Hs	1.6	Hc	0.035

**Table 2:** Operating bandwidth and fractional bandwidth of each absorption peak (A ≥ 0.9).

Freq (GHz)	90% Bandwidth Range (GHz)	Absolute BW (GHz)	Fractional BW (%)
2.178	2.10–2.24	0.14	6.4
5.484	5.43–5.57	0.14	2.6
8.391	8.32–8.51	0.19	2.2
11.811	11.72–11.96	0.24	2.0
15.858	15.70–16.10	0.40	2.5
18.689	18.55–18.90	0.35	1.9

**Table 3:** Resonant mode identification and operating mechanism of the proposed absorber.

Freq. (GHz)	Mode	Active Region	Physical Mechanism
2.178	EDM	Outer square ring	Strong surface current loop produces electric field confinement, resulting in a primary LC resonance
5.484	2nd-order LC	Inner square ring	Higher-order inductive current loop coupled with capacitive gaps
8.391	MDM	1st hexagonal ring	Anti-parallel surface currents form a closed magnetic loop enabling impedance matching
11.811	Hybrid E–M	2nd hexagonal ring	Coupled electric and magnetic fields generate a mixed resonance with enhanced absorption
15.858	Coupled mode	All rings	Strong near-field mutual coupling among resonators enables broadband loss channeling
18.689	High-order dipole	Diamond rings	Localized edge currents and fringing capacitance dominate the resonance

**Table 4:** Comparison of the present work with previously reported comparable works.

Ref	No. of Absorption Bands	Resonance Freq (GHz)	Unit Cell Size (mm)	Thickness of Unit Cell (mm)	Absorptivity (%)
[23]	4	2.24, 2.87, 4.30 and 5.87	20	1.5	96
[25]	5	5.4, 8.7, 14.53, 15.25, 15.68	20	12	99
[26]	3	3.4, 9.6 and 14	10	1	99.9
[27]	3	3.36, 3.95 and 10.48	10	1.6	99
[28]	3	8.5, 13.5 and 17	8	0.4	99.9
This work	6	2.17, 5.48, 8.39, 11.81, 15.85, and 18.68	12.5	1.6	99.9

**Table 5:** Mapping between absorber resonances and common EMI sources in metallic enclosures.

Peak (GHz)	Interference Sources	Expected Effect in Enclosure
2.178	Industrial devices, RFID, IoT nodes	Reduction of enclosure re-radiation and leakage
5.484	Wi-Fi and ISM-band interference	Mitigation of internal standing waves and signal distortion
8.391, 11.811	High-speed digital harmonics, radar leakage	Improved EMC performance and reduced cross-coupling
15.858, 18.689	Satellite front-end and test instrumentation	Suppression of reflective noise at higher microwave frequencies

## Data Availability

All data generated or analysed during this study are included in this published article.
